# Amino Acid Catabolism: An Overlooked Area of Metabolism

**DOI:** 10.3390/nu15153378

**Published:** 2023-07-29

**Authors:** Nimbe Torres, Sandra Tobón-Cornejo, Laura A. Velazquez-Villegas, Lilia G. Noriega, Gabriela Alemán-Escondrillas, Armando R. Tovar

**Affiliations:** Departamento de Fisiología de la Nutrición, Instituto Nacional de Ciencias Médicas y Nutrición Salvador Zubirán, Vasco de Quiroga No 15. Col Belisario Domínguez-Sección XVI, Tlalpan, Mexico City 14080, Mexico; nimbe.torrest@incmnsz.mx (N.T.); sandra.tobonc@incmnsz.mx (S.T.-C.); laura.velazquezv@incmnsz.mx (L.A.V.-V.); lilia.noriegal@incmnsz.mx (L.G.N.); gabriela.alemane@incmnsz.mx (G.A.-E.)

**Keywords:** amino acid catabolism, gut microbiota, gene transcription, epigenetics, immune response, obesity, diabetes, thermogenesis

## Abstract

Amino acids have been extensively studied in nutrition, mainly as key elements for maintaining optimal protein synthesis in the body as well as precursors of various nitrogen-containing compounds. However, it is now known that amino acid catabolism is an important element for the metabolic control of different biological processes, although it is still a developing field to have a deeper understanding of its biological implications. The mechanisms involved in the regulation of amino acid catabolism now include the contribution of the gut microbiota to amino acid oxidation and metabolite generation in the intestine, the molecular mechanisms of transcriptional control, and the participation of specific miRNAs involved in the regulation of amino acid degrading enzymes. In addition, molecules derived from amino acid catabolism play a role in metabolism as they are used in the epigenetic regulation of many genes. Thus, this review aims to examine the mechanisms of amino acid catabolism and to support the idea that this process is associated with the immune response, abnormalities during obesity, in particular insulin resistance, and the regulation of thermogenesis.

## 1. Introduction

**Figure 1:**
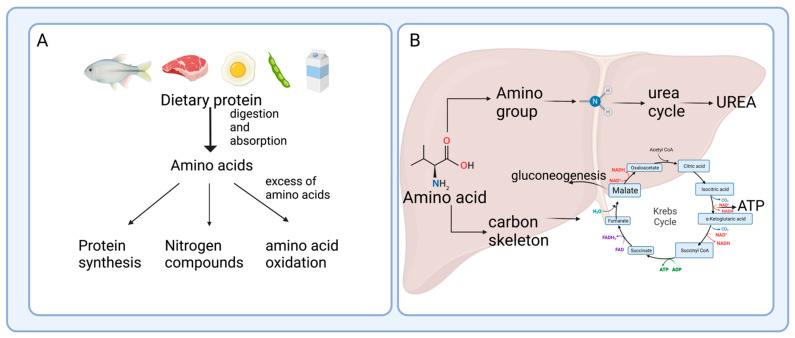
Amino acid catabolism in the liver. (**A**) Amino acids are used for protein synthesis and the generation of nitrogenous compounds. In the case of an excess in the consumption of amino acids, they are oxidized and utilized as energy fuel. (**B**) Amino acid catabolism in the liver involves several degrading processes, including the removal and transfer of the amino group, deamination, and transamination. Some amino acids undergo several degrading processes, like decarboxylation and dehydrogenation. The amino group enters the urea cycle for excretion. The carbon skeleton can be used for glucose formation by gluconeogenesis or for ATP production in the Krebs cycle, followed by the respiratory chain.

Amino acids are used to synthesize body proteins and serve as precursors of many molecules, including neurotransmitters, hormones, purines, pyrimidines, and some vitamins, among others. Upon fasting or when amino acids are ingested in excess of the amounts required, their catabolism serves as an energy source. When amino acids are used for energy production, they undergo the loss of their amino groups; their remaining carbon skeletons have two primary fates: conversion into glucose through gluconeogenesis or oxidation to synthesize ATP. For this reason, there must be a balance between the synthesis and continuous degradation of proteins in the body to ensure the maintenance of optimally functioning proteins. In the steady state, protein synthesis is equal to degradation, and neither the related transcription of amino acid degrading enzymes nor the metabolites generated from these processes are affected [1]. In addition, when the body protein reserve is altered due to increased protein degradation, as occurs, for instance, in the case of cachexia, vertebrates actively oxidize endogenous amino acids from the turnover of body proteins, similar to when ingested proteins exceed protein requirements (Figure 1A).

The catabolic pathways of most amino acids take place mainly in the liver, according to the large body of evidence reported throughout the development of metabolic biochemistry, with the exception of the branched-chain amino acids (BCAA), leucine, valine, and isoleucine [2]. The α-amino nitrogen atoms removed from amino acids during their oxidative degradation are ultimately excreted in the urine as urea to prevent the potential toxicity of a high amount of circulating ammonium. In the catabolism of most amino acids (Figure 1B), the removal of the α-amino group constitutes the first stage and occurs via two major enzymatic pathways: transamination and oxidative deamination. Then, as a second stage, the carbon skeletons of the amino acids are channeled into the tricarboxylic acid cycle to obtain energy in the form of ATP, and some serve as substrates for the gluconeogenic pathway [3]. However, new studies have shown that hepatic catabolism of amino acids is not their only fate. In other cell types or tissues, the catabolism of some amino acids plays a regulatory role that was not initially recognized. The complexity of the molecular mechanisms, as well as the potential metabolic implications associated with the regulation of amino acid catabolism in different physiological and pathological conditions, have been scarcely reviewed. Thus, this review aims to understand the mechanisms of regulation of amino acid catabolism and to highlight the influence of some metabolites, products of their degradation, on the epigenetic regulation of several genes and the involvement of the intestinal microbiota in amino acid catabolism. Further consequences of alterations in these new mechanisms of amino acid catabolism have implications on the immune response during obesity, type 2 diabetes, and thermogenesis, as will be described.

## 2. Enterohepatic Axis of Amino Acid Catabolism

After protein digestion, amino acids are absorbed and used not only by intestinal cells but also by the resident bacteria; once they have passed through the gut, a fraction of the amino acids are transferred to the bloodstream to reach the liver [4]. Several studies have shown active amino acid utilization and turnover in the enterohepatic axis, which is key for several physiological processes, including the immune response [5], bacteria metabolism, and generation of end-products, where the intestine and the gut microbiota play an important role [6].

### 2.1. Protein Hydrolysis and Amino Acid Absorption in the Gut

**Figure 2:**
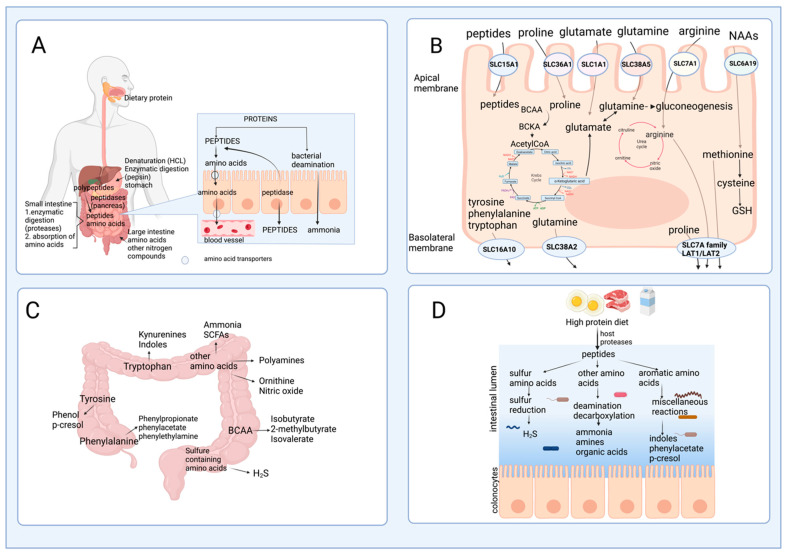
Protein hydrolysis, amino acid absorption, and catabolism in the gastrointestinal tract. (**A**) Upon reaching the stomach, proteins undergo a denaturation process caused by hydrochloric acid and pepsin to generate polypeptides [25]. In the small intestine, proteases and peptidases, released from the pancreas, continue the hydrolyzation of polypeptides, generating smaller peptides and free amino acids. Enterocytes in the small intestine also secrete peptidases that complete the hydrolysis of some peptides. (**B**) Amino acids are translocated from the intestinal lumen to the circulation by specific amino acid transporters (Solute Carriers Transporters (SLC)), expressed in the apical and basolateral membranes of the enterocytes [11]. (**C**) The bioavailability of amino acids and (**D**) protein consumed can generate multiple nitrogen-derived compounds or contribute to the generation of polyamines, nitric oxide, short-chain fatty acids, and ammonia, among others.

After passing through the esophagus and gastric lumen, dietary protein is hydrolyzed in the small intestine by the proteases and peptidases, including trypsin, chymotrypsin, or carboxypeptidases released by the pancreas [7]. In the intestinal mucosa, an intense renewal of protein occurs at approximately 50% per day in humans, indicating that amino acids are key effectors of gut protein turnover [8]. Many studies have focused on two amino acids, glutamine and arginine, in protein synthesis; however, amino acid catabolism in the intestine has not been extensively documented [8]. The brush border cells of the intestinal mucosa release some peptidases that are used for protein hydrolysis; these enzymes are more active at neutral to alkaline pH, which is reached in the small intestine, to catalyze the hydrolysis of dietary protein to produce amino acids and peptides that are actively absorbed by the enterocytes [9]. Although the process is quite efficient, some nitrogenated products are not taken up by the small intestine, pass through the ileocecal junction, and can be found in the large intestine [10]. Figure 2A outlines this process of protein digestion and absorption of peptides and amino acids, showing its passage through the gastrointestinal system.

### 2.2. Amino Acid Catabolism in the Intestine

Amino acids and some peptides found in the intestinal lumen after digestion can be delivered to the portal vein due to the presence of specific transporters known as solute carriers (SCL) in the brush border or apical membrane (SLC1A1, SLC6A19, SLC7A1, SLC38A5, SLC36A1, SLC15A1 transporters), and in the basolateral membrane of the enterocytes (members of the SLC7A family and SCL16A10, SLC38A2 transporters) [11] (Figure 2B). However, a large number of dietary amino acids, particularly BCAA, are partially catabolized in the gut since branched-chain aminotransferase (BCAT), the first enzyme in the degradation of the BCAA leucine, valine, and isoleucine, has been reported to be present in the gut [12]. Almost all dietary glutamate, aspartate, and approximately 30–70% of BCAA, glutamine, proline, lysine, threonine, methionine, and phenylalanine are metabolized in the small intestine of mammals, including humans [13]. These data suggest that several amino acid degrading enzymes (AADEs) are expressed in enterocytes. It has been shown that cells of the small intestinal mucosa express lysine α-ketoglutarate reductase in pigs [14]; methionine transamination [15] and glutamine transaminase K in rats [16]; threonine dehydrogenase in pigs [17]; BCAA transaminase and branched-chain α-keto acid (BCKA) dehydrogenase in humans and pigs [2,18]; and proline oxidase in pigs and rats [19]. Also, several enzymes for arginine and glutamine degradation are present, including arginase II, phosphate-dependent glutaminase (PDG), carbamoylphosphate synthase II (glutamine) (CPS-II), glutamate-oxaloacetate transaminase (GOT), glutamate-pyruvate transaminase (GPT), pyrroline-5-carboxylate (P5C) synthase, ornithine aminotransferase (OAT), P5C reductase, ornithine carbamoyltransferase (OCT), carbamoylphosphate synthase I (ammonia) (CPS-I), argininosuccinate synthase (ASS), argininosuccinate lyase (ASL), and ornithine decarboxylase (ODC) [20,21,22].

In the colonic lumen, colonocytes can use glutamine as a fuel substrate and catabolize arginine into ornithine and nitric oxide [23]. The conversion of arginine into ornithine significantly increases its capacity in response to an elevated protein intake, which attenuates the rising ammonia concentration in the blood if the urea cycle requires more ornithine [24].

### 2.3. The Role of Gut Microbiota in Amino Acid Catabolism and Generation of End-Products

Studies in germ-free animals reveal that gut bacteria alter the distribution of free amino acids in the gastrointestinal tract, indicating the role of the bacteria in the synthesis or degradation of amino acids. Interestingly, the amount of dietary protein and the dietary protein source modify the gut microbiota [26]. Consequently, this may suggest that the gut microbiota affects the bioavailability of amino acids to the host, depending on the taxonomy of intestinal bacteria. In addition, although short-chain fatty acids (SCFA) are the primary end product of carbohydrate fermentation, many amino acids formed by reductive deamination by bacteria can be precursors of SCFA. Certain bacteria in the small intestine, including *Klebsiella* spp., *Escherichia coli*, *Streptococcus* spp., *Succinivibrio dextrinosolvens*, *Mitsuokella* spp., and *Anaerovibrio lipolytica*, can also utilize protein digestive products and play an essential role in nitrogen recycling [27,28].

Moreover, bacteria species such as *Prevotella ruminicola*, *Butyrivibrio fibrisolvens*, *Mitsuokella multiacidas*, and *Streptococcus bovis* can secrete active dipeptidyl peptidase and dipeptidase that contribute to protein digestion and absorption of amino acids in the gut [29]. Ammonia is released daily by bacteria through the deamination of amino acids and, to a lesser extent, through urea hydrolysis catalyzed by bacterial urease activity. Up to 3.5–4.0 g of ammonia are released daily in the gut. Ammonia can be used by the bacteria for their metabolism and protein synthesis, absorbed by the colonocytes, transformed into urea in the liver, and excreted in urine [30]. It has been suggested that bacteria in the small intestine may participate in the catabolism of some essential amino acids and modulate the bioavailability of amino acids and their end-products in the systemic circulation of animals and humans. A previous study using conventional and germ-free rats with ^15^N incorporation into body lysine demonstrated that the N-lysine measured in the host came mainly from bacteria, indicating that amino acids can be exchanged between the host and the microbiota [31].

Although the digestion of dietary protein followed by amino acid absorption is a very efficient process in the small intestine, some nitrogenous material may escape digestion and be transferred into the large intestine. Undigested peptides and amino acids are usually not absorbed by the colonocytes but are fermented by gut microbes into intermediate metabolites and other end-products (Figure 2C), and this will depend on the amount of dietary protein consumed (Figure 2D). Some bacteria genera with proteolytic activity in the large intestine are Bacteroides, Propionibacterium, Streptococcus, Fusobacterium, Clostridium, and Lactobacillus [10,32]. Bacterial degradation of aromatic amino acids in the colonic lumen results in the production of phenolic and indolic compounds, ammonia, polyamines, and hydrogen sulfide [33,34]. Moreover, the catabolism of amino acids in these cells produces multiple metabolites, such as p-cresol from tyrosine, phenylpropionate, phenylacetate from phenylalanine, and indole skatole from tryptophan [30]. Phenolic compounds are primarily absorbed from the colon, detoxified in the colon mucosa and the liver by glucuronide and sulfate conjugation, and excreted in the urine. Decarboxylation of amino acids results in the appearance of amines in the gut. Monoamine and diamine oxidases present in the gut mucosa detoxify the amines produced by the gut microbiota [30].

## 3. Mechanisms of Regulation of Amino Acid Oxidation

Amino acid catabolism involves several metabolic pathways specific to a particular group of amino acids, involving multiple enzymes, some of which are essential for regulating the flux of metabolites in these pathways and are known as amino acid degrading enzymes (AADEs). AADEs are mainly located in the liver but are now known to be found in other extrahepatic tissues; they are specific for the utilization of each amino acid, and the transcription of the genes encoding them is highly regulated [35]. The study of the molecular mechanisms of regulation of AADEs had a significant breakthrough in the late 1980s and during the 1990s, when the transcriptional regulation of many AADEs by diet and various hormones was elucidated. These investigations provided sufficient information to understand certain key aspects of transcriptional control of AADEs that we now know are highly conserved among the genes of AADEs; however, recent studies have enriched previous knowledge by involving the participation of new players on the scene. Here we describe significant advances in the transcriptional regulation of AADE by diet, gut microbiota metabolites, and various hormones. We also provide recent evidence demonstrating that AADEs are potential targets of miRNAs. Finally, we highlight the importance of amino acid catabolism in epigenetic regulation mechanisms and cell reprogramming.

### 3.1. Regulation by the Gut Microbiota

Amino acids metabolized by bacteria present in the gut contribute to the survival and proliferation of these bacteria, but also provide amino acids and key metabolites to the host [36]. The generation of these metabolites can also indirectly modulate amino acid degradation in the intestine. For example, it is known that SCFA downregulates the expression of indoleamine 2,3-dioxygenase (IDO-1), a key enzyme involved in tryptophan degradation through the kynurenine pathway, in intestinal epithelial cells [37].

Dietary protein supply can also regulate amino acid catabolism indirectly via changes in the gut microbiota (Figure 2D). The consumption of a high-protein diet impacts the composition of the gut microbiota, favoring the accumulation of protein-fermenting bacteria and reducing the proportion of saccharolytic bacteria [38]. Moreover, the metabolites generated by the gut bacteria due to changes in the amount of dietary protein can indirectly affect the expression of AADE.

### 3.2. Transcriptional Regulation of Amino Acid Degrading Enzymes

As indicated above, each amino acid has a specific catabolic pathway regulated by key enzymes that play a central role in controlling the flux of the pathway metabolites [39], enzymes known as AADE, some of which are listed in Table 1. Early studies of amino acid catabolism showed that this process occurred mainly in the liver. However, it was later demonstrated that AADEs are present in different organs, particularly the liver, skeletal muscle, kidney, adipose tissue, intestine, and immune cells, among others [40,41,42,43,44]. Interestingly, the BCAA metabolism has some differences since the first step in the catabolism of BCAA is catalyzed by BCAT, an enzyme that is located in extrahepatic tissues, including skeletal muscle [45], the heart, kidney, brain, intestine, and adipose tissue, and in fetal rat liver [46]. The expression of these catabolic enzymes is highly responsive to the amount of dietary protein. Most of the AADEs are upregulated in the liver by the consumption of high-protein diets, indicating that the excess of protein will most likely be catabolized [44] because there is no amino acid reservoir in the body [42,47].

**Figure 3:**
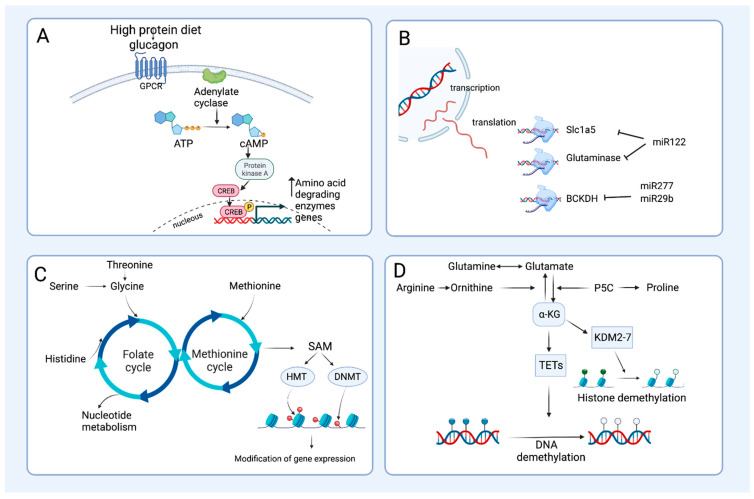
Transcriptional and post-transcriptional regulation of amino acid degrading enzymes and generation of amino acid derived metabolites as modulators of epigenetics. (**A**) The consumption of a high-protein diet induces the release of glucagon, which activates an intracellular Gαs-coupled proteinspathway after binding to its seven transmembrane receptors on the plasma membrane. This consequently increases cAMP levels via the activation of adenylate cyclase, which stimulates the activation of protein kinase A (PKA) and the cAMP response element-binding (CREB) protein. Phosphorylated CREB induces the transcription of several amino acid degrading enzymes in the liver, through its binding to specific response elements in their promoter regions. (**B**) Amino acid degrading enzymes can also be targets of some microRNAs (miRNAs), leading to their degradation and/or translational repression. Both miR277 and miR29b prevent the translation of proteins of the BCKDH enzyme complex [71,72], modifying the branched-chain amino acids/branched-chain alpha-keto acids ratio and activating TOR kinase, which regulates insulin signaling. miR122 inhibits the translation of mitochondrial glutaminase and its transporter, the Solute Carrier (SLC)1a5, thereby inhibiting glutaminolysis and enhancing gluconeogenesis in the liver [73]. (**C**) Amino acid degrading pathways provide metabolites that influence epigenetic modifications of chromatin that modulate gene expression. Methionine, threonine, serine, glycine, and histidine act as methyl donors; the generation of S-Adenosyl methionine (SAM) via the one-carbon metabolism plays a key role in epigenetic modification through DNA and histone methylation regulated by the activation of DNA methyltransferases (DNMT) and histone methyltransferases (HMT), respectively. (**D**) Catabolism of arginine, proline, histidine, glutamate, and glutamine can result in the generation of the epigenetic metabolite alpha-ketoglutarate (α-KG), which is used as a substrate for Jumonji C-domain lysine demethylases (KDM2-7) and ten-once translocation hydroxylases (TET1-3), enzymes responsible for histone and DNA demethylation, respectively. P5C: pyrroline-5-carboxylate.

Interestingly, the increased activity of these enzymes is due to an up regulation of AADE gene expression, as several studies have reported in mice or rats fed increasing amounts of dietary proteins showing an increment in the abundance of AADE mRNA in the liver, and the higher the amount of dietary proteins, the higher the expression of most of the enzymes [12,44,51]. Interestingly, when protein intake is above the protein requirement, there is also a significant increase in the abundance of BCAT in skeletal muscle [12]. After consuming high protein diets, glucagon levels increase, which stimulates the gene expression of AADE in the liver to catabolize the excess amino acids [51,52]. Similarly, during fasting, there is an increase in glucagon, which, through cAMP, also increases the expression of the AADE gene, leading to increased ATP synthesis and gluconeogenesis [53] (Figure 3A). In addition to glucagon, the fasting state increases the circulating levels of glucocorticoids [54]. Thus, transcriptional regulation studies have been conducted to establish the mechanism of action of glucocorticoids (dexamethasone) and glucagon on AADE gene expression. The first studies using the gene of tryptophan dioxygenase 2 (TDO2) showed that deletion of the 5′ region of its promoter causes the loss of regulation by dexamethasone [55]. Two response elements have been identified for glucocorticoids (GRE) at −450 bp and −1.2 kb upstream of the transcription start site of the TDO2 gene, which have common sequences GGTACANNNTGTT [56]. Further studies on other AADEs such as serine dehydratase (SDS) revealed that the −133 to −33 bp region is essential for its gene expression since a complete loss of activity is observed when this region is deleted, attributing the regulation to the transcription factors HNF1 and HNF4α [57]. It was later demonstrated that glucocorticoids and glucagon activate HNF4α [58]. In fact, recent studies indicate that the gene expression of the SDS gene is upregulated by the transcription factor HNF4α [59]. On the other hand, it was shown that two CREB response elements (CRE), designated CRE1 and CRE2, at approximately −3.5 kb from the transcription start site of the gene, also induced the expression of the SDS gene. Mutations of both CRE sites that are spliced partially decrease the activation of SDS gene transcription by cAMP, indicating that glucagon can directly stimulate the transcription rate of the SDS gene via protein kinase A (PKA). CRE sites are considered classic enhancers that positively stimulate the expression of SDS and other AADEs. Interestingly, dexamethasone can increase the binding of CREB to CRE sites by stimulating their phosphorylation [60]. Studies with another AADE, such as histidase (HAL), have revealed the presence of several response elements for various transcription factors, such as C/EBP, NF-IL6, HNF4α, progesterone and glucocorticoid receptors, and CREB in the regulatory region of its gene. The presence of CRE elements in the region between −340 and −815 bp reinforced the evidence that glucagon stimulates HAL gene expression via CREB [61]. In addition, stimulation with phorbol 12-myristate, 13-acetate (PMA) also activates the HAL promoter, indicating that gene expression of this catabolizing enzyme gene can also be stimulated via PKC [61]. Recently, HNF4α has been found to regulate the GLS2 promoter positively, and the response elements for HNF4 are conserved between human, mouse, and rabbit [62], response element also found in the SDS and HAL promoters [59,63]. Analysis of other AADE such as glutaminase 2 or tyrosine aminotransferase gene promoters, also provides evidence of the presence of response elements for CRE, HNF1, HNF-5, and GRE [64,65]. Interestingly, PPARα is known to promote proteasomal degradation of HNF4α, decreasing the transcription of AADE, but at the same time promoting the expression of fatty acid oxidation enzymes, resulting in a switch-off mechanism to prevent amino acid oxidation but activating the utilization of fatty acids as an energy source [63].

In addition, other conditions can induce the expression of specific AADEs. BCAA catabolism also increases in the fetal liver during gestation [46,66] and the mammary gland during lactation. In the last case, the high rate of blood flow to the mammary gland during lactation produces retention of BCAA in the gland, which exceeds the requirements for milk synthesis, stimulating the catabolism of BCAA, especially leucine [67]. The amino acid catabolism in the liver results in an increase in NH_4_^+^ production and an increase in the amino acid carbon skeletons. As a consequence, the enzymes of the urea cycle increase their expression with a high-protein diet [68] to eliminate the excess of NH_4_^+^. Other studies have shown that not only an excess of dietary protein induces the expression of these enzymes, but that prolonged fasting or the ingestion of very low concentrations of dietary protein increases muscle protein breakdown, releasing amino acids into the circulation, particularly glutamine and alanine, that are catabolized in the liver [54].

### 3.3. Regulation of Amino Acid Catabolizing Enzymes by miRNAs

In addition to transcriptional control, post-transcriptional control mechanisms can regulate the expression of AADE, particularly by some microRNAs (miRNAs). miRNAs are short 18–22 nucleotide, single-stranded RNA molecules that regulate gene expression by binding to 3′-UTR regions of mRNA target genes, leading to their degradation and/or translational repression [69]. Therefore, they can regulate and control metabolism by modifying gene expression. The first evidence suggesting miRNAs can regulate amino acid metabolism pathways was found after screening conserved 3′-UTR regions for potential miRNA targets in Drosophila melanogaster. It was found that miR-277 may be involved in regulating several enzymes of BCAA catabolism [70]. These results were validated when it was shown that miR-277 regulates BCAA catabolism by repressing the branched-chain α-ketoacid dehydrogenase (BCKDH) enzyme complex, thereby modifying the BCAA/BCKA ratio and activating TOR kinase, in turn affecting insulin signaling. It was also demonstrated that if miR277 is either expressed constitutively or inhibited, lifespan is shortened, especially when flies are fed high-protein/low carbohydrate diets, and dietary restriction attenuates these observations [71]. There is limited information in mammals, but it has been shown that miR29b prevents translation of the dihydrolipoamide branched-chain acyltransferase component of the BCKDH complex in HEK293 cell culture [72]. Elevated levels of circulating BCAA have been associated with the risk of obesity and type 2 diabetes, so it could be of great value to study whether miR29b expression is modified in these conditions.

Another example of the importance of miRNAs in the regulation of AADE is that of miR-122, which regulates glutamine metabolism in the liver by inhibiting mitochondrial glutaminase (Gls) and its transporter, Slc1a5, thereby inhibiting glutaminolysis and enhancing gluconeogenesis in humans and mice [73]. Since many tumors are highly dependent on glutamine as an energy source [74], targeting miR-122 for cancer treatment might be possible. This recent evidence shows the involvement of miRNAs in the regulation of the translation of proteins involved in amino acid catabolism [75], which is summarized in Figure 3B.

### 3.4. Amino Acid Catabolism and Epigenetics, Tissue Plasticity and Cellular Reprogramming

Nutrients can regulate the metabolic activities of living organisms through epigenetic mechanisms, including DNA methylation, histone modification, and RNA regulation [76]. Amino acid degradation pathways provide epigenetic metabolites that influence these processes. For example, some amino acids, particularly, methionine, glycine, histidine, and serine, act as dietary methyl donors and play a key role in epigenetic modification through DNA and protein methylation and one-carbon metabolism in the body [77] (Figure 3C). In addition, catabolism of arginine, proline, histidine, glutamate, and glutamine can result in the formation of alpha-ketoglutarate (α-KG), an epigenetic metabolite that is used as a substrate for enzymes responsible for epigenetic modifications of chromatin that modulate gene expression [78]. Among these enzymes, α-KG is utilized by Jumonji C-domain lysine demethylases (KDM2-7) [79], which are considered the major histone demethylases; and by ten-once translocation hydroxylases (TET1-3), which are involved in DNA demethylation and catalyze the oxidative decarboxylation of α-KG to produce 5-hydroxymethylcytosine (5-hmC) [80,81] (Figure 3D). In addition, some enzymes involved in amino acid degradation can be considered epigenetic regulators; this is the case of proline dehydrogenase, located in the mitochondrial inner membrane, which converts proline to pyrroline-5-carboxylate (P5C) and provides electrons to the electron transport chain [82]. This enzyme is under the regulation of p53 and PPARα, and its activation promotes the generation of ROS, which in turn generates epigenetic changes to induce the expression of the genetic program of antioxidant enzymes, cell cycle arrest, and autophagy [83,84].

Moreover, amino acid degradation produces downstream metabolites that are critical for embryonic stem cell (ESC) function and differentiation. Evidence from several laboratories reports that differentiation and self-renewal of mouse ESCs are dependent on proline catabolism. Likewise, catabolism of either threonine (mouse) or methionine (human) generates molecules used in histone methylation and acetylation involved in ESC differentiation and growth [85].

## 4. Amino Acid Catabolism in Health and Disease

Amino acid catabolism provides metabolites that serve as substrates or are responsible for activating signaling pathways involved in the physiology of different organs. For example, the regulation of amino acid catabolism is key for the immune system since its functions depend on an adequate supply of amino acids, and individual amino acids and their metabolites can affect immune responses [5]. Similarly, amino acid catabolism dysregulation can contribute to the development, or progression of pathological processes involved in the presence of insulin resistance [86]. Here we provide evidence for the importance of amino acid turnover and catabolism in health and disease, opening the landscape to new mechanisms regulated by amino acids and their metabolites beyond their known functions for protein synthesis and energy fuel.

### 4.1. Amino Acid Catabolism and Immunity

All cells, including those involved in immune responses, depend on nutrient availability to maintain their functionality [87]. When there is an inflammatory or antigenic cue, immune cells need more amino acids to remain viable and respond accordingly, so they must adapt rapidly to any shortage of amino acids [88]. This adaptation suggests that any modification in amino acid (and other nutrient) metabolism will also affect the immune response in different ways, depending on the specific nutrient and energy requirements of the cells and, thus, their function [89,90]. In other words, each immune cell’s microenvironment will be directly related to their response to nutrient availability. Both the innate and adaptive immune systems require an adequate supply of amino acids to synthesize molecules such as histamine, glutathione, and nitric oxide, among others, but especially for immunoglobulins and cytokine activation, as well as for antibody production through mTOR signaling by BCAA [91]. Therefore, individual amino acids and their metabolites can affect immune responses.

Besides BCAA, the AADE that have been mainly studied for their response to inflammation are those involved in tryptophan and arginine catabolism, such as tryptophan 2,3-dioxygenase (TDO), IDO1, the arginase isoforms (ARG1, ARG2), and the inducible nitric oxide synthase (iNOS), respectively [92].

#### 4.1.1. Tryptophan Catabolism

TDO and IDO catalyze the cleavage of the C2–C3 bond of the indolic ring of L-tryptophan to form N-formyl-kynurenine [93]. Noteworthy, this pathway is essential for NAD^+^ synthesis, which is necessary to control ATP production and macrophage activation and function. Although both enzymes catalyze the same reaction, they differ in expression and function [94]. TDO is mainly expressed in the liver and is induced by tryptophan, glucocorticoids, and kynurenine. Because it catabolizes excess dietary tryptophan, it may indirectly control serotonin production [95], which, among other things, can modulate cytokine release and leucocyte recruitment during inflammation responses [96]. Also, it has been shown that active TDO is highly expressed in tumor cells, and its expression contributes to tumoral immune resistance [97].

**Figure 4:**
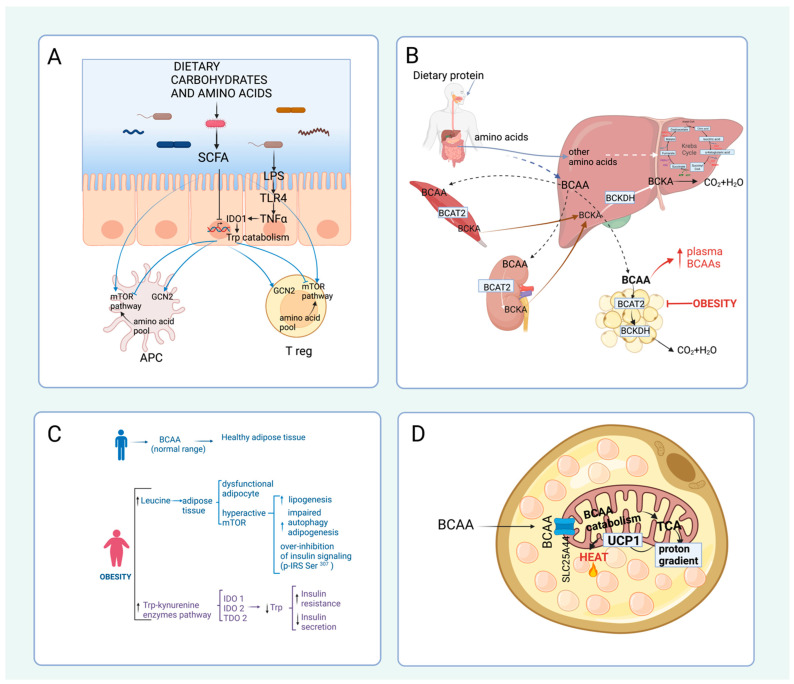
Influence of amino acid catabolism in physiopathological processes. (**A**) Individual amino acids and their metabolites can affect immune responses. In the intestinal epithelium, short-chain fatty acids (SCFAs) produced by bacteria from dietary carbohydrates and/or amino acids downregulate the expression of the tryptophan catabolism enzyme indoleamine 2,3-dioxygenase (IDO-1). In contrast, lipopolysaccharide (LPS) and TNF-α (tumor necrosis factor-a) generated by Gram-negative bacteria induce its expression. Tryptophan (Trp) catabolism in the gut can influence the amino acid pool. (**B**) Transamination of the branched-chain amino acids (BCAA) by BCAT2 is extrahepatic for branched-chain alpha-keto acids (BCKA) production. The liver mitochondrial BCKDH complex catalyzes the oxidative decarboxylation of BCKA. (**C**) Increased levels of BCAA are found in obesity and type 2 diabetes. BCAA metabolism generates some intermediates of tricarboxylic acid (TCA), leading to an increase in fatty acid oxidation that could decrease glucose utilization in the skeletal muscle. mTORC1 activation activates lipogenesis and adipogenesis and inhibits autophagy and insulin signaling by inactivating the insulin receptor substrate 1 (IRS1) in the white adipose tissue. Decreased Trp levels due to an increase in the expression of enzymes (IDO1, IDO2, and TDO2) of the Trp-Kynurenine pathway are associated with decreased insulin secretion and insulin resistance. (**D**) Amino acid catabolism also plays a role in the induction of thermogenesis in the brown adipose tissue (BAT). BAT mitochondria express BCAT2 and BCKDH and can use BCAA as an energy source to increase thermogenesis. BCAA are transported into the mitochondria by the SLC25A44 transporter to generate reduced intermediates that pump protons in the respiratory chain to produce heat through uncoupling protein 1 (UCP1).

IDO1, the rate-limiting step of tryptophan degradation, is extrahepatic and is induced by lipopolysaccharide (LPS), TNF-α (tumor necrosis factor-α), and IFN-γ (interferon-γ) [98,99] (Figure 4A). The latter induces host cells to degrade tryptophan in response to a bacterial infection, therefore blocking the growth of bacteria by limiting this amino acid [100]. Moreover, upon interferon induction, some antigen-presenting cells, including macrophages and dendritic cells, also express IDO1 to deplete amino acids in the intestinal lumen and regulate the immune response in the intestinal barrier [92]. It has also been shown that IDO expression in dendritic cells can increase regulatory T cells (Tregs) through activation of the aryl hydrocarbon receptor (AHR) [101]; however, whether the increase in Tregs is due to a reduction in tryptophan concentration or by kynurenine production is still a matter of debate.

#### 4.1.2. Arginine Catabolism 

L-arginine is a common substrate for arginase-1 (Arg1) and inducible nitric oxide synthase (iNOS); thus, its regulation is essential for proper cellular functioning [102]. Arg1 produces urea and L-ornithine from L-arginine, while iNOS converts the latter into L-citrulline, producing nitric oxide (NO) [103]. Macrophages are part of the innate immune system and respond to invading bacteria, viruses, fungi, and even cancer cells, changing their phenotype [104]. The two main phenotypes are classically activated macrophages (M1) and alternatively activated macrophages (M2) [105]. M1 macrophages are activated by LPS and IFN-γ producing pro-inflammatory cytokines, while M2 macrophages are activated by IL4, IL13, and lactic acid and produce anti-inflammatory cytokines [106,107]. Interestingly, among other differences between both phenotypes, M1 macrophages express iNOS to produce NO as a microbicidal agent, and M2 macrophages express Arg-1 to increase polyamine synthesis from ornithine and promote wound healing and tissue fibrosis [108,109]. These examples show that amino acid catabolism is key for the energy and functional demands of the intestinal epithelium, such as the regulation of the intestinal barrier and immune response [110]. A more detailed description of the involvement of amino acids and their catabolism in immune responses is reviewed elsewhere [5,88,92,110,111].

### 4.2. Amino Acid Catabolism in Obesity and Diabetes

An adequate amino acid catabolism is crucial to maintaining plasma amino acid concentration. Disturbances in plasma amino acid concentration have been associated with alterations in individual health [86]. In a cross-sectional study with young adults, it has been observed that subjects with obesity have higher levels of alanine, aspartate, cysteine, ornithine, phenylalanine, proline, and tyrosine and lower levels of glycine, ornithine, and serine compared to normal weight subjects [112]. Furthermore, subjects with insulin resistance (IR) (defined as HOMA > 2.5) have higher levels of arginine, alanine, aspartate, isoleucine, leucine, phenylalanine, proline, tyrosine, taurine, and valine than subjects without IR [112]. In addition, increased levels of BCAA and aromatic amino acids are associated with a five-fold increased risk of developing type 2 diabetes [113,114]. BCAAs play important general roles in the body, including regulation of protein synthesis through mTOR and as a source of energy during exercise, which have been extensively reviewed elsewhere [115]. However, there is significant controversy as to whether altered plasma concentrations of amino acids, especially BCAA, are a cause or consequence of obesity or insulin resistance. Unlike the rest of the amino acids, as mentioned above, the first enzymatic reaction of BCAA catabolism is extrahepatic, with muscle, kidney, and adipose tissue as the principal organs for BCAA transamination (Figure 4B). Notably, BCAA catabolism is mainly impaired in adipose tissue during obesity. Branched-chain aminotransferase 2 (BCAT2), an isoform of BCAT found in mitochondria, and BCKDH activity are reduced in the adipose tissue of mice and rats with genetic or diet-induced obesity [116,117,118,119]. In subjects with obesity, BCAT2 and BCKDH expression is reduced mainly in visceral adipose tissue [43,117]. Nevertheless, further research is needed to clarify the mechanisms responsible for the decreased expression and activity of BCAT2 and BCKDH in adipose tissue during obesity.

The alteration of BCAA catabolism has physiological consequences in two aspects. First, leucine is likely the most potent mTORC1 activator among all amino acids. In physiological conditions, mTORC1 activation inhibits autophagy, activates adipogenesis and lipogenesis in white adipose tissue, and inhibits insulin signaling through the inactivation of the insulin receptor substrate 1 (IRS1) (Figure 4C). Thus, high leucine concentrations could induce an overactivation of mTORC1, leading to insulin resistance and, thus, an increase in glucose levels [120,121]. The use of rapamycin, a known mTORC1 inhibitor, prevents glucose intolerance induced by a diet high in fat and BCAA [122].

Furthermore, leucine catabolism contributes 30% to the acetyl-CoA lipogenic pool [123]. and acetyl-CoA can be converted into malonyl-CoA by acetyl-CoA carboxylase (ACC). Malonyl-CoA is the preferred substrate of fatty acid synthase (FAS). Thus, leucine is extensively incorporated into the lipid fraction of functional adipocytes. Incorporation that is significantly reduced in adipocytes from high-fat fed rats [118]. In addition to fatty acids, the carbon skeleton of leucine could be a substrate for synthesizing phospholipids and cholesterol. An increase in cholesterol-synthetizing enzymes has been demonstrated, which use 3-hydroxy-3-methylglutaryl-CoA, an intermediate of leucine catabolism, as a substrate during adipogenesis [124]. Taken together, this evidence suggests that decreased catabolism of BCAA could reduce the production and storage of FA and cholesterol in adipose tissue, affecting its functionality.

Second, a reduction in the BCAA catabolic enzymes causes an inefficient production of anaplerotic substrates from BCAA, causing suboptimal activity of the Krebs cycle [125,126]. These results have important implications for the understanding of metabolic inflexibility. Until now, metabolic flexibility has only been assessed in terms of how glucose affects lipid metabolism and vice versa. However, new evidence suggests that amino acid catabolism needs to be evaluated in terms of metabolic flexibility because amino acid availability and catabolic amino acid metabolites can also affect lipid and carbohydrate metabolism, as in the model proposed by Muoio, where chronic overfeeding causes a mitochondrial blockade affecting glucose, fatty acid, and BCAA oxidation [127].

Although progress has been made in understanding the participation of BCAA catabolism in the maintenance of adipocyte function, further research is needed to understand the role of catabolism of other amino acids such as aspartate, proline, tyrosine, and tryptophan, among others, that can modify mitochondrial activity and therefore metabolic flexibility in the adipocyte.

In addition to BCAAs, tryptophan (Trp) is another essential amino acid for humans, [128]. Consumed tryptophan is mainly used for protein synthesis; however, this amino acid can be used in about 5% of cases as a precursor for the synthesis of serotonin, N-acetyl serotonin, and melatonin [129]. Interestingly, a part of free tryptophan is also catabolized through the tryptophan-kynurenine pathway [130]. Recent studies have found that certain metabolites of tryptophan catabolism participate in the development of T2D [131]. A significant association between low plasma Trp concentrations has been reported in obese subjects with metabolic syndrome, in whom insulin resistance is common [132]. Evidence suggests that metabolites of the kynurenine pathway increase with insulin resistance before the clinical manifestation of hyperglycemia [133]. Gene expression of tryptophan catabolism limiting enzymes to kynurenine, such as indolamine 2,3-dioxygenase 1 (IDO1), indolamine 2,3-dioxygenase 2 (IDO2), and tryptophan 2,3-dioxygenase (TDO2), is shown to increase in patients with T2D [134]. A possible mechanism by which metabolites of the Trp-Kynurenine pathway contribute to the development of insulin resistance includes the possible formation of chelate complexes between xanthurenic acid and insulin, which are indistinguishable from free insulin but have ∼50% less activity than insulin [135]. However, studies are still needed to determine the importance of the regulation of gene expression by the step-limiting enzyme of the Trp- Kynurenine pathway in the pathogenesis of diabetes or its complications. On the other hand, Trp and phenylalanine interact with the GPR142 receptor, increasing insulin secretion and the incretins GIP and GLP-1, improving circulating glucose levels [136], suggesting that the decrease in Trp during obesity will decrease insulin secretion and contribute to the progression to T2D of subjects with obesity.

### 4.3. Amino Acid Catabolism and Thermogenesis

In addition to white adipose tissue, it has been demonstrated that BAT may play an essential role in the control of body thermogenesis [137]. This effect is in part mediated by the presence of the uncoupling protein 1 (UCP1), which can use the proton gradient generated in the mitochondria by the respiratory chain in order to produce heat [138]. The energy sources used by BAT mitochondria are fatty acids and glucose [139]. Interestingly, recent evidence has demonstrated that BAT mitochondria can use BCAA amino acids as an energy source to increase thermogenesis (Figure 4D). BAT is now known to express enzymes of the amino acid catabolic pathways for BCAA, including BCAT2 and BCKDH. Under conditions of cold exposure, where thermogenesis is upregulated, there is a significantly increased uptake of BCAA by BAT, particularly valine and leucine. The importance of BCAA oxidation for thermogenesis has been demonstrated since the absence of BCKDH expression in BAT impairs energy homeostasis, especially during cold exposure. BCAA oxidation occurs in the mitochondria, and it is known that BCAAs are transported into the mitochondria by the SLC25A44 transporter. Surprisingly, the deletion of SLC25A44 impairs BAT thermogenesis [140].

As previously mentioned, white adipose tissue plays an important role in the utilization of BCAAs, which is associated with improving insulin sensitivity. However, now BAT is also considered an essential catabolic organ of these amino acids, decreasing circulating levels of BCAA that are associated with increased insulin sensitivity.

## 5. Conclusions

Amino acid catabolism has been considered in nutrition as a mechanism used by the body to eliminate excess circulating amino acids due to high-protein intake or a catabolic state of the organism, as occurs during fasting or in specific pathologies. However, research in nutrition and metabolism has shown that amino acid catabolism plays an essential role in different metabolic processes, even though it has long been overlooked. It is now important to consider it as a key element of metabolism whose abnormalities may be associated with the development of several chronic diseases. Amino acid catabolism enzymes have been found in different cell types or organs and perform specific functions, generating end-products that can be used for ATP synthesis and gluconeogenesis. However, its role is now known to have additional functions of great relevance, such as the regulation of the immune response, the development of insulin resistance, and even body thermogenesis. It is important to comprehensively consider not only the utilization of dietary amino acids as precursors for the synthesis of proteins and other nitrogenous compounds, but also that their utilization by the catabolism machinery generates important metabolites that have an impact on the regulation of gene expression that directly or indirectly impacts the fine regulation of numerous processes in various cell types of various organs or tissues. Interestingly, new important players in the orchestration of amino acid catabolism have been discovered, particularly the gut microbiota. Thus, the field of nutrition must take into more profound and more applicative consideration the effects of amino acid catabolism. In particular, the catabolism of BCAAs needs to be studied in greater depth, as current evidence strongly suggests its implications in various metabolic processes, and it is therefore important to conduct more clinical trials to use this information in the development of dietary guidelines or therapeutic strategies involved in the future of personalized nutrition.

## Figures and Tables

**Table 1:** Amino acid degrading enzymes and main organ locations in humans, rats and mice.

Amino Acid (1-Letter)	AADE	NameAADE	Tissue Expression Humans [48]	Tissue Expression Rat [49]	Tissue Expression Mouse [50]
Alanine (A)	Glutamic pyruvate transaminase	Gpt	Liver, Kidney, fat, colon, duodenum small intestine, stomach	Liver, lung muscle, thymus, uterus	Liver, duodenum, large intestine, subcutaneous fat pad, stomach
Arginine (R)	Arginase 1	Arg1	Liver, skyn, bone marrow	Liver, lung, testes, uterus	Liver, lung, ovary
Asparagine (N)	Asparaginase	Aspg	Liver, kidney, heart, ovary, colon, sky, stomach, lung	Liver, kidney, lung. Adrenal, testes	Liver, kidney, mammary gland, colon, large intestine
Aspartic acid (D)	glutamic-oxaloacetic transaminase 1	Got1	Heart, liver, kidney, brain, duodenum, colon, small intestine	Heart, muscle, brain, liver, lung	Heart, liver, kidney, cerebellum, cortex
Cysteine (C)	Cysteine dioxygenase 1	Cdo1	Liver, fat, placenta, testis, brain, lung	Liver, thymus, adrenal, lung	Liver, genital fat, mammary gland, subcutaneous fat pad
Glutamic acid (E)	Glutamate dehydrogenase 1	Glud1	Liver, kidney, prostate, brain, small intestine, stomach, adrenal (16)	Liver, kidney, brain, uterus, muscle, heart	Liver, kidney, small intestine, adrenal
Glutamine (Q)	Glutaminase	Gls	Liver, kidney, small intestine, brain, duodenum, lung	Kidney, brain, adrenal, lung, muscle, testes, thymus, liver	CNS E18, Cortex, frontal lobe, cerebelum, thymus
Glycine (G)	Serine hydroximethyl transferase 1	Shmt1	Kidney, liver, fat, duodenum, small intestine, esophagus, thyroid	Kidney, liver, adrenal, testes, thymus	Liver, kidney, placenta, testis
Histidine (H)	Histidine ammonia lyase	Hal	Liver, skin, bone marrow, appendix, spleen.	Liver, lung, testes, uterus.	Liver, genital fat pad, placenta
Lysine (K)	Glutaryl-CoA dehydrogenase	Gcdh	Liver, kidney, ovary, fat, heart	Kidney, liver, adrenal, lung, muscle	Liver, kidney, adrenal, heart
Methionine (M)	Methionine adenosyl transferase	Mat1a	Liver, pancreas, ovary, skin, testis	Liver, lung, uterus, adrenal	Liver
Phenylalanine (F)	Phenylalanine hidroxylase	Pah	Liver, kidney, gall bladder	Liver, kidney, lung, testes, uterus	Kidney, liver
Proline (P)	Proline dehydrogenase	Prodh	Small intestine, skin, lung, duodenum, brain, kidney	Liver, kidney, heart, thymus, brain	Kidney, liver, large intestine, small intestine, genital, duodenum
Serine (S)	Serine dehydratase	Sds	Liver, stomach, brain	Liver, kidney, testes	Liver, heart
Threonine (T)	Serine dehydratase like	sdsl	Kidney, liver, thyroid, adrenal, colon duodenum	Testes, liver, kidney, brain, uterus	Adrenal, duodenum, ovary, colon
Tryptophan (W)	Tryptophan 2,3-dioxygenase	Tdo2	Liver, appendix, urinary bladder, small intestine	Liver, lung, uterus	Liver, placenta
	Aminocarboxymuconate semialdehyde decarboxylase	Acmsd	Kidney, liver, gall bladder	Kidney, liver	Kidney, liver
Tyrosine (Y)	Tyrosine aminotransferase	Tat	Liver	Liver, kidney, colon, large intestine	Liver, lung, uterus, adrenal

## Data Availability

Not applicable.
